# Evolving Tale of TCPs: New Paradigms and Old Lacunae

**DOI:** 10.3389/fpls.2017.00479

**Published:** 2017-04-03

**Authors:** Namrata Dhaka, Vasudha Bhardwaj, Manoj K. Sharma, Rita Sharma

**Affiliations:** ^1^Crop Genetics & Informatics Group, School of Computational and Integrative SciencesJawaharlal Nehru University, New Delhi, India; ^2^Crop Genetics & Informatics Group, School of BiotechnologyJawaharlal Nehru University, New Delhi, India

**Keywords:** gene regulation, plant development, plant morphology, stress response, TCP domain, transcription factor

## Abstract

*Teosinte Branched1/Cycloidea/Proliferating cell factors* (TCP) genes are key mediators of genetic innovations underlying morphological novelties, stress adaptation, and evolution of immune response in plants. They have a remarkable ability to integrate and translate diverse endogenous, and environmental signals with high fidelity. Compilation of studies, aimed at elucidating the mechanism of TCP functions, shows that it takes an amalgamation and interplay of several different factors, regulatory processes and pathways, instead of individual components, to achieve the incredible functional diversity and specificity, demonstrated by TCP proteins. Through this minireview, we provide a brief description of key structural features and molecular components, known so far, that operate this conglomerate, and highlight the important conceptual challenges and lacunae in TCP research.

## Introduction

TCP (*Teosinte Branched1*/*Cycloidea*/*Proliferating cell factors*) is a plant-specific family of transcription factors (TFs), with the earliest members reported in fresh water charophyte algae (Cubas et al., 1999a; Navaud et al., 2007). TCP proteins are characterized by a non-canonical beta helix-loop-helix (bHLH) domain, known as TCP domain. Although, TCP proteins have little homology with bHLH TFs and bind to DNA elements distinct from those recognized by bHLH TFs; the DNA contacting residues and mechanism of binding seem to be conserved in both the families (Kosugi and Ohashi, 1997). Aggarwal et al. (2010) suggested divergent evolution of TCP domain from the bHLH domain by insertion of a short stretch in the basic region thereby, splitting the long helix into two.

TCP family comprises six genes each in bryophyte species, *Selaginella* and *Physcomitrella* (Navaud et al., 2007). Whereas, the size of this family in angiosperms ranges from 12 in the orchid, *Orchis italica* (De Paolo et al., 2015) to more than 60 in tobacco (Chen et al., 2016) and cotton (Ma et al., 2016).

Multiple sequence alignment revealed two major classes of the TCP family viz., classes I and II. The residue composition in the DNA-binding TCP domain and, supplementary motifs confer specific characteristics to the members of both the classes. Some of the notable differences include a four-amino-acid deletion in the basic region of the class I TCPs and presence of additional motifs, such as glutamic acid-cysteine-glutamic acid (ECE) stretch and/or arginine-rich R-domain in a subset of class II proteins (Cubas et al., 1999a; Navaud et al., 2007). Class II further comprises two distinct subclasses namely, CINCINNATA (CIN) and CYCLOIDEA/TEOSINTE BRANCHED 1 (CYC/TB1). CIN clade is ubiquitous, whereas, CYC/TB1 is restricted to angiosperms and has undergone extensive duplications and diversification giving rise to three different clades: CYC1, CYC2, and CYC3 (Howarth and Donoghue, 2006).

## TCP Genes are Key Mediators of Morphological Innovations, Stress Adaptations, and Plant Immunity Evolution

The studies done in experimentally tractable *Arabidopsis*, and several non-model plant species revealed that TCPs have played key role in generating novel morphologies during plant evolution (Martin-Trillo and Cubas, 2010; Manassero et al., 2013; Li, 2015). Since structural features play important role in determining protein functions, distinctive functions have been associated with members of each class. For example, class I genes (*TCP6*–*9, 11, 14*–*16, 19*–*23*) mostly act as positive regulators of cell division in diverse biological processes ranging from seed germination, leaf and floral organ development, gametophyte development and senescence (Martin-Trillo and Cubas, 2010; Li, 2015; Nicolas and Cubas, 2016). A recent study involving expression of a dominant repressor form of *TCP16* demonstrated the ability of class I genes in modulating meristematic programs and differentiation state of the plant cells (Uberti-Manassero et al., 2016).

Class I TCP genes of rice have been mainly implicated in stress adaptation. *PCF2* of rice affects salinity tolerance by positively regulating expression of a Na^+^/H^+^ antiporter gene, *OsNHX1* (Almeida et al., 2017). Whereas, *PCF5* and *6* are involved in drought plus salinity, and cold stress tolerance, respectively (Luo et al., 2012; Wang et al., 2014). *OsTCP19*, on the other hand, influences both development and abiotic stress tolerance by manipulating abscisic acid (ABA) signaling network (Mukhopadhyay and Tyagi, 2015). Also, mesocotyl elongation in response to darkness in rice has been associated with expression of *OsTCP15* (Hu et al., 2014).

Members of the CYC/TB1 clade of class II (*TCP1, 12*, and *18*) are mainly involved in regulating shoot branching, floral transition, organ identity, and development. A mutation in *TB1* locus is responsible for the domestication of maize from its wild ancestor, teosinte (Doebley et al., 1995, 1997). Expression of another maize TCP gene *BRANCHED ANGLE DEFECTIVE 1* in a grass-specific structure (pulvinus), between main stem and lateral branches of inflorescence, influences lateral branch angle and inflorescence architecture (Bai et al., 2012). The recent studies in non-model systems, cucumber and melon, revealed the role of CYC/TB1 genes in determining tendril identity, as well (Mizuno et al., 2015; Wang C. et al., 2015). A rare single nucleotide polymorphism in a TCP gene *TEN* is responsible for the tendril-less phenotype in cucumber (Wang S. et al., 2015).

Among the three subgroups of CYC clade, *CYC1* genes have retained *TB1*-like functions across different taxa in regulating branching. Characterization of *TB1* orthologs from monocots, such as rice (*Fine culm1*/*OsTB1*), barley (*INTERMEDIUM-C*), *Sorghum* (*SbTB1*), and switchgrass (*PvTB1*) and dicots, such as *Arabidopsis* (*BRC1* and *BRC2*), pea (*PsBRC1*), and tomato (*SlBRC1*) indicate conserved role of this gene in negatively regulating axillary bud outgrowth across both the lineages of angiosperms (Takeda et al., 2003; Kebrom et al., 2006; Aguilar-Martínez et al., 2007; Ramsay et al., 2011; Braun et al., 2012; Nicolas et al., 2015; Xu et al., 2016). Duplication and differential expression of *CYC2* genes have played a key role in the evolution of symmetry across different lineages of the angiosperms (Luo et al., 1996; Reeves and Olmstead, 2003; Specht and Howarth, 2015; Yang et al., 2015). *CYC* ortholog of rice, *RETARDED PALEA 1* (*REP1*), also played a key role in regulating floral zygomorphy (Yuan et al., 2009). Whereas, *CYC3* genes in *Arabidopsis* have been reported to play a minor role in branching in both vegetative and floral organs (Finlayson, 2007).

Genes belonging to CIN clade (*TCP2*–*5, 10, 13, 17*, and *24*) of class II have been mainly implicated in regulating flowering time, floral organ development, leaf development and senescence, and morphogenesis of lateral organs (Nath et al., 2003; Palatnik et al., 2003; Koyama et al., 2007; Schommer et al., 2008; Ballester et al., 2015; Yang et al., 2015). Some of the more recent roles reported include regulation of secondary cell wall thickening in roots and floral organs of *Arabidopsis* (Wang H. et al., 2015) and ovule development in *Phalaenopsis equestris* (Lin et al., 2016). Although in angiosperms, only *CYC*/*TB1* genes have been implicating in branching, a recent study in *Physcomitrella patens* revealed a role of CIN gene *PpTCP5* in determining sporangia architecture by negatively regulating branching (Ortiz-Ramírez et al., 2016). These results indicate regulation of branching as an ancient role of class II TCPs.

Furthermore, members of both the classes are targeted by pathogens to manipulate host defense. An effector SECRETED AY-WB PROTEIN 11 (SAP11), produced by aster yellows phytoplasma, binds and destabilizes TCP4 thereby, leading to reduced jasmonic acid (JA) synthesis, increased plant susceptibility and survival rate of the insect vector (Sugio et al., 2011, 2014). TCP13, 14, and 19 of *Arabidopsis* are also directly targeted by pathogen effectors to elicit effector-triggered susceptibility. Whereas, TCP8, 14, and 15 interact with Suppressor Of rps4-RLD1 (SRFR1), a negative regulator of effector-triggered immunity to influence plant susceptibility (Kim et al., 2014). Recently, Zhang et al. (2016) showed that infection with viral pathogen, rice ragged stunt virus (RRSV) in rice leads to increased accumulation of miR319-targeted TCP genes, decreased JA levels and increased plant susceptibility. The biotrophic pathogens, however, may be benefited from the activation of JA-dependent responses. A recent study showed that *Pseudomonas syringae* type III effector, HopBB1 interacts with *Arabidopsis* TCP14 and targets it to proteasome-mediated degradation. Consequently, *TCP14*-regulated subset of JA response genes are de-repressed thereby, promoting pathogen virulence (Yang et al., 2017).

## Binding Site and Mechanism of Action

TCP proteins modulate gene expression by directly binding to the regulatory regions of their target genes. Previous studies have reported overlapping but specific binding sites of classes I and II proteins. Viola et al. (2012) showed that presence of glycine or aspartic acid at positions 11 and 15 in classes I and II proteins, respectively, determines their binding preference. However, changes in residue composition at other positions can also influence the DNA-binding preferences of TCP proteins (Viola et al., 2011). For example, class I TCP protein, TCP11, has distinct DNA binding specificity due to presence of threonine residue at position 15, occupied by arginine in most of the other TCP proteins (Viola et al., 2011). Biochemical studies in *Arabidopsis* revealed that redox state of the cell can also influence binding ability of class I TCP proteins (Viola et al., 2013). Oxidation of a conserved cysteine residue at position 20 (cys-20) in these proteins leads to formation of intermolecular disulfide bonds and covalently linked homodimers that cannot bind target DNA. The effect of *Arabidopsis* TCP15 on anthocyanin accumulation is lost after prolonged exposure to high light intensity due to oxidation of cys-20 (Viola et al., 2016).

Presence of co-regulators may be imperative for the regulatory activity of TCPs. For example, a WD repeat-containing protein, LIGHT-REGULATED WD1 (LWD1) acts as a coactivator of TCP20 and 22 in regulating expression of morning gene *CIRCADIAN CLOCK ASSOCIATED1* (*CCA1*) in *Arabidopsis* (Wu et al., 2016). Although TCP20 and 22 can bind to regulatory element in *CCA1* promoter, even in the absence of LWDs, overexpression of *TCP20*/*22* in *lwd1lwd2* double mutant fails to activate *CCA1* expression (Wu et al., 2016). Whether concomitant binding of TCPs and LWDs leads to any shifts in conformational state of TCPs is yet to be determined.

Several TCPs act as modulators of hormone biosynthesis, transport and signal transduction (Lopez et al., 2015; Nicolas and Cubas, 2016). A recent review summarizes crosstalk between TCPs and, biosynthesis and signaling of hormones viz., gibberellins, cytokinins, ABA, JA, brassinosteroids, strigolactones, and auxins (Nicolas and Cubas, 2016).

Cell/tissue-type or developmental stage-specific expression of members of same/different class seems to assist them in fine tuning the hormone production and balance. For example, *TCP20* of class I suppresses expression of *LIPOXYGENASE2* (*LOX2*), a key enzyme involved in JA biosynthesis in young leaves, whereas, *TCP4* of class II promotes *LOX2* expression thereby, promoting JA biosynthesis and senescence in mature leaves (Danisman et al., 2012). The same gene, *TCP4*, however, suppresses *LOX2* expression in floral tissues (Rubio-Somoza and Weigel, 2013).

The role of TCPs in regulation of hormone activity may be indirect by interacting with regulators of hormone biosynthesis and response as exemplified by interaction of OsTCP19 with ABA INSENSITIVE4 and of OsTB1 with OsMADS57 (Nicolas and Cubas, 2016). Alternatively, TCPs may directly bind to the promoters of key genes involved in hormone biosynthesis as exemplified by regulation of DWARF4 by TCP1 and, regulation of LOX2 by TCP4/20 (Nicolas and Cubas, 2016). A recent study showed that YUCCA5, an enzyme involved in auxin biosynthesis, is direct target of TCP4 (Challa et al., 2016).

TCP proteins also regulate transcription of the non-coding RNAs that in turn target genes involved in hormonal signaling. For example, TCP4 directly regulates miR167a that targets auxin response factors, ARF6 and 8, involved in JA biosynthesis (Nagpal et al., 2005; Wu et al., 2006).

**FIGURE 1:**
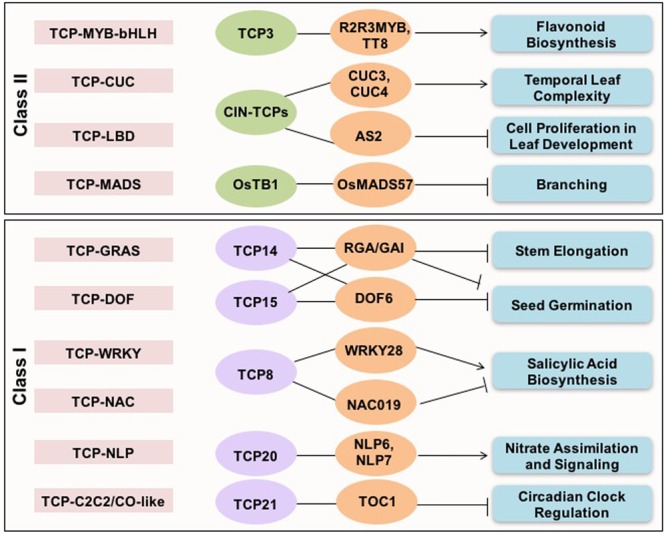
**Cross-family transcription factor interactions shown by TCP proteins.** The cross-family TF interactions exhibited by both classes I and II TCP proteins, and associated biological pathways are presented.

Analysis of cross-family TF interactions showed that TCPs exhibit high range of connectivity with members of other TF families (Bemer et al., 2017). Synergistic interactions between members of different TF families binding to different *cis*-elements in the targeted genes imply a combinatorial effect on target gene expression (**Figure 1**). TCP21 (CHE) of *Arabidopsis* interacts with C2C2/CO-like family component of circadian clock, TIMING OF CAB EXPRESSION1 (TOC1) during circadian regulation (Pruneda-Paz et al., 2009). The direct interaction between an *Arabidopsis* DOF TF, DOF6, and TCP14 affects seed germination (Rueda-Romero et al., 2012). CIN-TCPs interact with LBD domain containing ASYMMETRIC LEAVES 2 (AS2) TF to suppress KNOX gene expression during leaf development in *Arabidopsis* (Li et al., 2012). Similarly, the ternary complex between TCP, MYB, and bHLH family TFs (TCP3-R2R3MYB-TT8) is involved in regulating flavonoid biosynthesis and auxin response (Li and Zachgo, 2013). An interaction between MADS-box protein OsMADS57 and OsTB1 has been shown to modulate tillering in rice (Guo et al., 2013). Whereas, the interaction between TCP14 of *Arabidopsis* with GRAS domain containing DELLA proteins in inflorescence apical meristems determines plant height (Daviere et al., 2014). Interaction between CUC family TFs, CUC2 and 3 and, TCP4, regulates age-dependent leaf complexity in *Arabidopsis* (Rubio-Somoza et al., 2014).

The choice of interaction partners also contributes to the functional diversity and specificity. For example, TCP8 may activate or repress *ISOCHORISMATE SYNTHASE 1* (*ICS1*), a key gene involved in salicylic acid biosynthesis, by interacting with the transcriptional activators, WRKY28 and SAR DEFICIENT 1 or the NAC family repressor NAC109, respectively (Wang X. et al., 2015). Interactions between TCP20 and NIN-like TFs has been recently demonstrated to regulate nitrate assimilation and signaling (Guan et al., 2017). Overall, these studies highlight that TCP proteins are at the center of plant molecular networks and control diverse range of processes and signaling networks by recruiting specific interaction partners. Presence of intrinsically disordered region gives them extra flexibility to interact with diverse range of partners and make higher order complexes (Valsecchi et al., 2013).

## Regulation of TCPs

The ability of TCPs to orchestrate plant response to both internal cues such as developmental signals and circadian rhythms; and diverse environmental factors such as light quality, nutrient availability, oxidative stress, etc., requires precise spatial and temporal control of their activity. Current research shows that the regulation of TCPs acts at several steps including transcription, mRNA stability, and post-translational modifications.

Regulation of gene expression includes a wide array of mechanisms. The spatial/temporal expression of TCP genes is directly associated with specific morphological phenotype or physiological response. For example, differential expression of *GhCYC2* in *Gerbera* controls morphological differentiation of flower types along the radial axis of inflorescence (Broholm et al., 2008). Changes in the regulatory region of *TB1* due to two transposable element insertions are responsible for its differential expression and domestication of maize (Zhou et al., 2011).

Alternative splicing also plays significant role in regulating gene expression. In potato, quality of light (R:FR) determines the ratio of two isoforms of a TCP gene *BRC1a*, only one of which is localized to nucleus and acts as a transcriptional activator (Nicolas et al., 2015). Transcriptional regulation by epigenetic mechanisms has also been demonstrated in TCPs. Differential methylation pattern in *CYC* orthologs resulted in differential expression of the gene causing dorsoventral asymmetry in flowers of *Linaria vulgaris* (Cubas et al., 1999b).

Role of non-coding microRNAs in post-transcriptional regulation of TCPs involved in flowering time and leaf morphogenesis is well-documented (Palatnik et al., 2003; Schommer et al., 2012; Spanudakis and Jackson, 2014). Both *PCF5* and *6* of rice, involved in abiotic stress tolerance, are direct targets of miR319 (Luo et al., 2012; Wang et al., 2014). Downregulation of miR319-targeted *TCP4*, in response to sulfur dioxide exposure in *Arabidopsis*, reinforce the role of miRNAs in environmental regulation of TCPs (Li et al., 2016).

The final control comes at the level of post-translational modifications. These affect the activity and stability of the protein. Steiner et al. (2016) reported that regulation of TCP14 by SPINDLY, a Ser and Thr *O*-linked *N*-acetylglucosamine (*O*-GlcNAc) transferase (OGT), prevents its proteolysis. Similarly, ubiquitin receptor proteins, DA1 and DA1-related proteins (DAR1 and DAR2), physically interact with TCP14 and 15, and affect their ubiquitination and stability (Peng et al., 2015). Ubiquitination sites have also been found on class I TCPs, TCP8 and 22, whereas, Ser-211 in TCP8 is phosphorylated (Valsecchi et al., 2013; Walton et al., 2016).

## Key Challenges and Outlook

TCP genes appear to play central role in the biological signaling networks by interacting with many molecular and signaling components. These features not only make them ideal candidates to investigate the mechanism of combinatorial gene expression and hormonal crosstalk in plants, but also suggest them as promising targets for engineering crop plants. For this, a thorough understanding of their mechanism of action is imperative. Most of the functional genomic studies with TCPs are impeded by lack of three-dimensional structure, high level of genetic redundancy and lack of sufficient *in vivo* studies to identify *in planta* interaction partners and other regulatory components.

The theoretical predictions based on bHLH structure can be misleading. Deciphering three-dimensional structures of representative TCP proteins is of fundamental importance to gain mechanistic understanding of their functions. To cope with redundancy in TF genes, Hiratsu et al. (2003) developed a novel approach using a chimeric repressor gene-silencing technology (CRES-T), in which a TF is fused to the EAR-motif repression domain (SRDX) that dominantly represses the transcription of its target genes even in the presence of functionally redundant TFs (Mitsuda et al., 2011). Several authors have successfully used this technology to gain insights into TCP gene functions in *Arabidopsis* (Koyama et al., 2007; Guo et al., 2010; Aguilar-Martinez and Sinha, 2013). However, this technology cannot be used to decipher functions of essential genes. Danisman et al. (2012) used a bioinformatics approach to integrate data generated using pair-wise protein-protein interactions, phylogeny and expression profiling to predict functionally redundant TCP genes in *Arabidopsis*. Authors also validated one of the novel pairs, TCP19-TCP20, that functions redundantly in the leaf development. However, the interactions reported in their study are not immune to limitation of yeast two-hybrid technology. Due to high auto-activation capacity of class I TCP proteins, most of the connections were reported among class II TCP proteins. *In planta* studies during temporal stages of development and in response to pathogen infection or abiotic stresses would be required to precisely determine the interaction dynamics of TCP proteins.

Another interesting aspect of TCP genes is the predominant presence of introns in their UTRs (Francis et al., 2016). How these intron sequences influence gene expression, mRNA stability, or translational efficiency in TCPs remains unexplored.

Furthermore, although miR319-mediated regulation of CIN genes in both dicot and monocot species is well-documented, none of the TCP genes in *Physocmitrella, Selaginella*, and *Marchantia polymorpha* have a recognizable miR319 binding site (Axtell and Bowman, 2008; Schommer et al., 2012; Flores-Sandoval et al., 2016). Future studies will clarify if gain of miR319 targeting site has any role in the functional evolution of CIN genes in higher plants.

Furthermore, most of the earlier studies aimed at characterizing TCP gene functions focused on the model system, *Arabidopsis.* Although the TCP gene functions are now beginning to be elucidated in non-model systems as well (**Table 1**), this area of TCP research still needs momentum.

**Table 1:** Teosinte Branched1/Cycloidea/Proliferating cell factors (TCP) proteins characterized from non-model systems and their roles.

	Species	Gene	Function	Reference
**Dicots**	*Brassica rapa*	*BrpTCP4*	miR319a-regulated, regulates transition from round to cylindrical head shape	Mao et al., 2014
		*BrTCP24*	Suppresses growth of plant cells in Chinese cabbage	Gao et al., 2016
	*Cucumis melon*	*CmTCP1*	Involved in development of tendrils from lateral shoots	Mizuno et al., 2015
	*Cucumis sativus*	*TEN*	Causal gene for rare variation of tendril-less phenotype	Wang S. et al., 2015
	*Gerbera hybrida*	*GhCYC2*	A gradient of *GhCYC2* expression correlates with flower type specification along inflorescence axis	Broholm et al., 2008
	*Gossypium hirsutum*	*GhTCP14*	Regulates auxin-mediated development of cotton fiber cells	Wang et al., 2013
	*Ipomoea nil*	*InTCP4*	miR319-regulated, affect floral initiation, flower development and cotyledon senescence	Glazińska et al., 2014
	*Pisum sativum*	*PsBRC1*	Regulates shoot branching putatively in response to cytokinin and strigolactone signaling	Braun et al., 2012
	*Solanum lycopersicon*	*LA (LANCEOLATE)*	miR319-regulated, involved in leaf margin development and compound leaf formation	Ori et al., 2007
		*SlBRC1b*	Suppresses shoot branching	Martín-Trillo et al., 2011
		*SlTCP14-2*	Target of pathogen effector CRN12_997 of *Phytophthora capsici* and prevents plant defense	Stam et al., 2013
	*Solanum tuberosum*	*BRC1a*	Involved in controlling lateral branching	Nicolas et al., 2015
**Monocots**	*Hordeum vulgare*	*INTERMEDIUM-C*	Regulate tillering and fertility of lateral spikelets	Ramsay et al., 2011
	*Oryza sativa*	*FC1 (FINE CULM1)*	Ortholog of maize *TB1* and mutants exhibit reduced plant height and increased tillering	Takeda et al., 2003
		*REP1 (RETARDED PALEA1)*	Controls palea development and floral zygomorphy	Yuan et al., 2009
		*OsTCP5*	Controls mesocotyl elongation in rice	Hu et al., 2014
		*OsTCP19*	Involved in salinity and drought tolerance	Mukhopadhyay and Tyagi, 2015
		*OsTCP21*	Involved in cold stress tolerance and plant defense response against rice ragged stunt virus (RRSV)	Wang et al., 2014; Zhang et al., 2016
		*PCF2*	Involved in salt stress tolerance	Almeida et al., 2017
		*PCF5*	Involved in drought and salinity stress tolerance	Luo et al., 2012
		*PCF6*	Involved in cold tolerance	Wang et al., 2014
	*Petunia hybrida*	*PhTCP3*	Regulates branching through strigolactone signaling	Revel et al., 2015
	*Phalaenopsis equestris*	*PePCF10*	Involved in leaf and ovule development	Lin et al., 2016
		*PeCIN8*	Regulates ovule, leaf and petal development	Lin et al., 2016
	*Sorghum bicolor*	*SbTB1*	Negatively regulates tillering by suppressing bud outgrowth	Kebrom et al., 2006
	*Switchgrass*	*PvTB1*	Negatively regulates tillering	Xu et al., 2016
	*Zea mays*	*BAD1*	Regulates inflorescence architecture by affecting lateral branch angle	Bai et al., 2012
		*TB1*	Negatively regulates tillering and promotes formation of female inflorescence	Doebley et al., 1997
**Bryophytes**	*Physcomitrella patens*	*PpTCP5*	Negatively regulates sporophyte branching	Ortiz-Ramírez et al., 2016

## Author Contributions

ND and RS conceptualized, prepared the framework and drafted the review. VB collected the data from the literature and helped in drafting the manuscript. MS contributed in preparing the framework and revising the article. All authors read and approved the article.

## Conflict of Interest Statement

The authors declare that the research was conducted in the absence of any commercial or financial relationships that could be construed as a potential conflict of interest.
